# Synergistic Reduction of Breast Cancer Cell Viability and Aggressiveness Through Dual Inhibition of APE1 Redox Function and STAT3 Signaling

**DOI:** 10.1002/cbin.70094

**Published:** 2025-10-15

**Authors:** Mariana Moreno de Sousa Rodrigues, Priscyanne Barreto Siqueira, Ana Clara Cavallo Dobao, Maria Eduarda Barbosa Mourão, Bruno Ricardo Barreto Pires, Adenilson de Souza da Fonseca, Ísis Salviano Soares de Amorim, Andre Luiz Mencalha

**Affiliations:** ^1^ Departamento de Biofísica e Biometria, Instituto de Biologia Roberto Alcântara Gomes, Laboratório de Biologia do Câncer Universidade do Estado do Rio de Janeiro Rio de Janeiro Brazil; ^2^ Departamento de Biofísica e Biometria, Instituto de Biologia Roberto Alcântara Gomes, Laboratório de Biofotônica Universidade do Estado do Rio de Janeiro Rio de Janeiro Brazil; ^3^ Instituto de Nutrição Josué de Castro, Laboratório de Alimentos Funcionais Universidade Federal do Rio de Janeiro Rio de Janeiro Brazil

**Keywords:** aggressiveness, APE1, breast cancer, STAT3

## Abstract

Aggressiveness and resistance to treatments are significant problems in cancer management. In this scenario, searching for new pharmacological targets for therapies is essential. The APE1 redox domain coactivates transcription factors that favor cancer malignancy. One of APE1's targets, STAT3, coordinates the transcription of genes involved in cancer hallmarks. However, the association between APE1 and STAT3 in the context of breast cancer cell survival and aggressiveness has not been previously characterized. Therefore, we investigated the role of the redox function of APE1 and STAT3 inhibitors in cell viability, proliferation, migration, invasion, and cell death. In addition, we verified the association between APE1 and STAT3 in breast cancer patient samples from TCGA and their relationship with proliferation and metastasis. Our results suggest that combined treatment with APE1 and STAT3 inhibitors can further synergistically reduce cell viability, proliferation, migration, and invasion, compared to treatment with inhibitors alone. Moreover, the APE1 and STAT3 activity levels positively correlated with proliferation and metastasis gene signatures. Thus, we suggest the APE1 redox domain and STAT3 as promising targets for new therapy strategies against breast cancer.

## Novelty and Impact

In this study, we first reported that APX2009 impairs STAT3 transcriptional activity in breast cancer cells, leading, in combination with Stattic, to a further decrease in the survival and aggressiveness of breast cancer cells in vitro, compared to treatments alone. Moreover, APE1 and STAT3 signatures are positively correlated in breast cancer data from TCGA and are positively correlated with proliferation and metastasis signatures. Therefore, dual‐targeting APE1 redox activity and STAT3 could be a promising new therapeutic approach against breast cancer.

**Abbreviations**
AP‐1: activator protein 1
CI: combination index
DMEM: Dulbecco's Modified Eagle Medium
DMSO: Dimethylsulfoxide
DNA: deoxyribonucleic acid
EMT: epithelial‐mesenchymal transition
FBS: Fetal Bovine Serum
HER‐2: human epidermal growth factor receptor 2
HIF‐ 1α: hypoxia‐inducible factor
NF‐κB: nuclear factor kappa B
RPMI: Roswell Park Memorial Institute medium
STAT3: signal transducer and activator of transcription 3
TCGA: The Cancer Genome Atlas Program

## 1 Introduction

Breast cancer is molecularly and cellularly heterogeneous, which varies disease prognosis, aggressiveness, and difficulty in cancer treatment management. Despite the existence of several breast cancer therapies, the disease is still at the top of the worldwide women's death list. The accelerated tumor cell proliferation and metastasis are the main factors that keep breast cancer a challenge. There are several emerging strategies to target cancer cells, including the inhibition of proteins related to cancer, which can be performed through silencing (Tang et al. 2022), direct inhibitors (Guo et al. 2022a), and decoy oligodeoxynucleotides (Johari et al. 2020; Mousazadeh et al. 2022; Rahmati et al. 2020). In this context, new pharmacological compounds may be evaluated to improve treatment efficacy (Wilkinson and Gathani 2022).

In this context, APE1 is a protein that has been demonstrated to be a relevant target due to its participation in DNA repair and transcriptional regulation pathways. Additionally, APE1 is frequently overexpressed in several cancers, including lungs, liver, bladder, and breast cancer, which suggests its role in cancer hallmarks (Malfatti et al. 2023). This protein contains two domains: the C‐terminal domain presents a deoxyribonucleic acid (DNA) repair function, and the N‐terminal redox function. The redox domain of APE1 is responsible for coactivating different transcription factors. Some transcription factors already determined are Signal transducer and activator of transcription 3 (STAT3), Hypoxia‐inducible factor (HIF‐1α), nuclear factor kappa B (NF‐κB), Activator protein 1 (AP‐1), and p53. These transcription factors act in different hallmarks of cancer, such as cell proliferation, inflammation, and angiogenesis, activation of invasion and metastasis, which promote tumor progression (Shah et al. 2017; Siqueira, de Sousa Rodrigues, et al. 2024)

Among these transcription factors, STAT3 participates in the JAK/STAT pathway, in which it is activated and regulates genes related to promoting essential processes for cancer, such as the regulation of cell proliferation and survival (Ma et al. 2020). STAT3 is also highly correlated with the tumor‐aggressive phenotype, contributing to a worse prognosis in cancer (Tolomeo and Cascio 2021). APE1 and STAT3 are highly expressed in cancers such as pancreatic, ovarian, and breast cancer (Banerjee and Resat 2016; Malfatti et al. 2023; Wen et al. 2016). APE1 and STAT3 could individually coordinate several cancer cells' aggressiveness phenotypes, such as proliferation, viability, cellular migration, and invasion (Guan et al. 2023; Siqueira, Rodrigues, et al. 2024). Although both APE1 and STAT3 are important proteins in tumor progression, the crosstalk between these proteins is still unknown in breast cancer and processes related to tumor aggressiveness.

**Figure 1:**
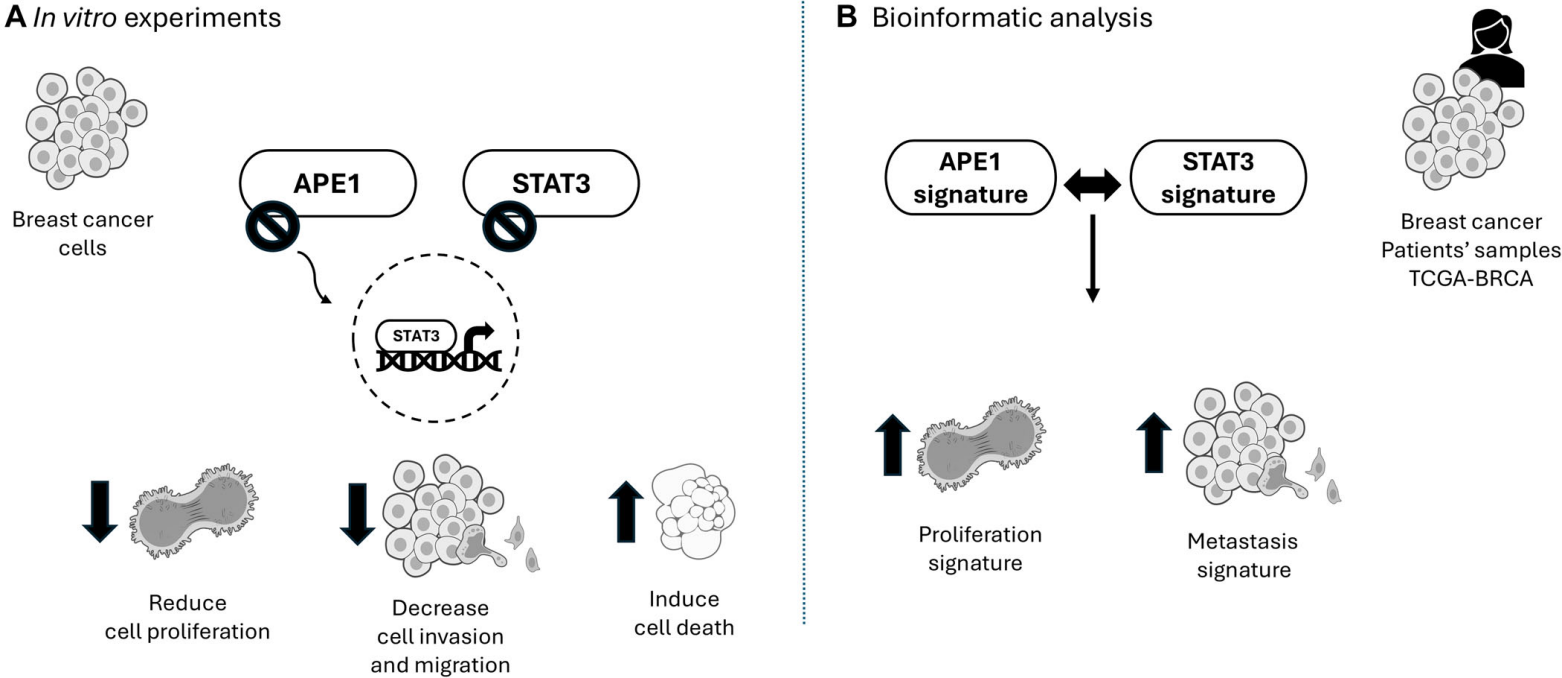
Schematic overview of the study's main findings. (A) In vitro experiments showed that the treatment with APX2009 can impair STAT3 transcriptional activity. Moreover, the combined treatment with redox APE1 and STAT3 inhibitors can synergistically reduce cell viability, proliferation, migration, and invasion, and induce cell death in MDA breast cancer cells. (B) Bioinformatic analysis of breast cancer patient samples from TCGA suggest a positive correlation between redox APE1 and STAT3 activity levels with proliferation and metastasis gene signatures.

Here, we investigated the role of APE1 redox activity in the transcriptional activity of STAT3 and their crosstalk on proliferation, migration, and invasion processes in breast cancer cells. We demonstrate that the inhibition of the APE1 redox domain decreases STAT3 transcriptional activity in breast cancer. Moreover, dual targeting of redox APE1 and STAT3 signaling reduces cell viability, proliferation, migration, invasion potential and induces cell death by apoptosis in breast cancer cells (Figure 1). Altogether, our data suggests that the crosstalk between APE1 and STAT3 is critically for breast cancer survival and aggressiveness, and their inhibition could be promising for the development of future therapies.

## 2 Materials and Methods

### 2.1 Cell Culture

MDA‐MB‐231 (HTB‐26, ATCC, RRID: CVCL_0062), a triple‐negative breast cancer cell line, was used as a breast cancer model with an increased aggressive phenotype. MCF‐7 (HTB22, ATCC, RRID: CVCL_0031), a Luminal A breast cancer cell line, was used as a breast cancer model with a low aggressive phenotype (Phannasil et al. 2023). MCF10A (CRL‐10317, ATCC, RRID: CVCL_0598) is a non‐tumoral breast cell line used as a control for cytotoxicity assay (Soule and McGrath 1991). MDA‐MB‐231 and MCF‐7 cell lines were cultured in Roswell Park Memorial Institute medium (RPMI) (ThermoFisher, 31800105) and Dulbecco's Modified Eagle Medium (DMEM) (ThermoFisher, 12800017), respectively, supplemented with 10% of Fetal Bovine Serum (FBS) (Cripion, FB0010S) and 1% of antibiotic and antimycotic (ThermoFisher, 15240062) at 37°C and 5% of CO2. MCF10A cells were cultured in DMEM and DMEM F12 (ThermoFisher, 12500062) (1:1) supplemented with 10% FBS, 1% of antibiotic and antimycotic, 10 µg/mL of insulin (Sigma‐Aldrich, I0516), 20 ng/mL of Epidermal Growth Factor (Sigma‐Aldrich, E5036), and 0.5 µg/mL of hydrocortisone (Novafarma), at 37°C and 5% of CO2 (Mou et al. 2025).

### 2.2 Inhibitors

Stattic (Sigma‐Aldrich, S7947) is a specific STAT3 inhibitor of STAT3 transcriptional activity (McMurray 2006; Rodrigues et al. 2024). APX2009 (Sigma‐Aldrich, SML1887) inhibits the redox domain of APE1 (Kelley et al. 2016). DMSO (Dimethylsulfoxide, MERCK, 102952) was used as a vehicle.

### 2.3 WST‐1 Cell Viability Assay

After 24 h of treatment with Stattic and/or APX2009, cell viability was analyzed using a WST‐1 (Roche, 5015944001) colorimetric assay (Coperchini et al. 2019; de Amorim et al. 2025). The cells were seeded in 96‐well plates (7 × 10³ cells/cm² for MDA‐MB‐231 and 12 × 10³ cells/cm² for MCF‐7) and cultured in a humidified incubator with 5% CO2 at 37°C. After cell growth, the cells were treated with 3, 6, 10, 30, 60, and 90 µM of Stattic, 0.8, 4, 10, 20, and 50 µM of APX2009, and concentrations of combined inhibitors, 30 and 60 µM of Stattic + 20 µM of APX2009. DMSO was used as a control. After 24 h of treatment, the cells were incubated with 10 µL of WST‐1 for 1 h at 37°C. The absorbance was obtained with a microplate reader (Polaris) at 450 nm. The percentage of viable cells was represented as a percentage of treated/untreated cells.

### 2.4 Combination and Selectivity Index

The Combination Index (CI) was calculated using Compusyn Software (Version 1.0), based on the effects that the inhibitors caused alone and combined in each cell line. A CI equal to 1 is considered additive, less than 1 is synergism, and greater than 1 is considered antagonism. This software also generates the isobologram of the combinations. In this graph, the line formed diagonally represents the additive effect of the compounds. Above this line, the effect of the combination is characterized as antagonism, and below the line, it is classified as synergism (Chou 2010). The selectivity index (SI) was calculated to analyze the selectivity of APX2009 and Stattic in cancer cells. For this aim, we used the formula: SI = (IC50 of MCF10A cells)/(IC50 of MDA‐MB‐231 or MCF‐7 cells) (Silva et al. 2021). The IC50, concentration required for the treatment to lead to a 50% reduction in cell viability, was calculated based on a sigmoidal regression curve using GraphPad Prism 8.0.2 software (Cho et al. 2022; Ramos et al. 2024).

### 2.5 Anchorage‐Dependent Colony Formation Assay

Anchorage‐dependent colony formation assay was used to analyze cells' clonogenic potential after treatment with Stattic and/or APX2009. The cells were seeded in 6‐well plates (4 × 10³ cells/well for MDA‐MB‐231 and 8 × 10³ cells/well for MCF‐7). After 4 h, the cells were treated with 0.3, 0.6, 1, and 3 µM of Stattic, 0.16, 0.8, and 4 µM of APX2009, and the combination of 0.3 and 0.6 µM of Stattic + 0.16 and 0.8 µM of APX2009. After 7 days of culture, the cells were fixed in 2 mL of ethanol 100% for 10 min and stained with 0.5% crystal violet in 20% ethanol for 10 min. Then, the cells were washed three times, and the crystal violet was eluted with 10% acetic acid (de Souza et al. 2013). 150 µL of each sample was used to measure the absorbance with a microplate reader (Polaris) at 595 nm, and images were acquired using a digital camera (Motorola Moto One Action).

### 2.6 AnnexinV/7‐AAD Staining by Flow Cytometry

The Annexin V‐FITC (ThermoFisher, A13199) and 7‐AAD (ThermoFisher, A1310) staining by flow cytometry was performed to analyze apoptosis and necrosis (Siqueira, Rodrigues, et al. 2024; Torcasio et al. 2024). The cells were seeded in a 6‐well plate (7 × 10³ cells/cm² for MDA‐MB‐231 and 12 × 10³ cells/cm² for MCF‐7) and cultured in a humidified incubator with 5% CO2 at 37°C. After cell growth, the cells were treated with 6, 10, and 30 µM of Stattic and 0.8, 4, and 20 µM of APX2009 for 24 h. Then, cells were trypsinized and washed with PBS. Next, 10^5^ cells were centrifuged, resuspended in 100 µL of Annexin V Binding Buffer (ThermoFisher, V13246), 2 µL of FITC Annexin V, and 2 µl of 7‐AAD Viability Staining Solution, and incubated for 15 min. After that, 400 µL of Annexin V Binding Buffer was added, and 10^4^ cells were analyzed by flow cytometry (FACSCalibur, BD Biosciences), using Floreada. io (https://floreada.io). One mM of Staurosporine (Sigma‐Aldrich, S6942) was used as a positive control for apoptosis, and Triton™ X‐100 0, 1% was used as a positive control for necrosis.

### 2.7 Wound Healing Assay

The wound healing assay was performed to analyze cell migration after treatment with Stattic and/or APX2009 (Siqueira, Rodrigues, et al. 2024; Xu et al. 2023). The cells were seeded in 6‐well plates (6 × 10^4^ cells/cm²) to form a cell monolayer at 90% confluence. After 24 h of growth for MDA‐MB‐231 and 48 h for MCF‐7, the medium with 10% FBS was removed and replaced with 1% FBS to prevent proliferation (Siqueira, Rodrigues, et al. 2024; Suh et al. 2022). After 4 h, the cell monolayers were wounded by scratching with a 200 µL plastic tip for MDA‐MB‐231 and with a 10 µL plastic tip for MCF‐7, the medium was replaced by a fresh medium with 1% FBS, and MDA‐MB‐231 cells were treated with 6 µM of Stattic or 0.8 and 4 µM of APX2009, while MCF‐7 cells were treated with 10 and 30 µM of Stattic or 4 and 20 µM of APX2009. These treatments were performed using nonlethal concentrations to avoid indirect effects on cell migration. Images were acquired using a digital camera (Motorola One Action) at 0 and 24 h after treatment, and the free area was quantified in pixels using ImageJ Fiji (NIH). The formula used to quantify was the ratio between the scratched area at 24 and 0 h. This value was decreased by 1 to quantify the cell's migration in relation to the free area (Bobadilla et al. 2019).

### 2.8 Matrigel Transwell Invasion Assay

Matrigel Transwell invasion assay was performed to analyze cell invasion after treatment with Stattic and/or APX2009 (Joo et al. 2023; Siqueira, Rodrigues, et al. 2024). The upper side of the transwell (8 μm pore size) chamber was coated with a Matrigel basement membrane‐like matrix (10 mg/mL) (Sigma‐Aldrich, CLS354234), where the cells were seeded (2 × 10^4^ cells/cm² for MDA‐MB‐231 and 2 × 105) and treated with 6 and 10 µM of Stattic or 0.8 and 4 µM of APX2009 for MDA‐MB‐231, while MCF‐7 were treated with 10 and 30 µM of Stattic or 4 and 20 µM of APX2009. The lower chamber of the transwell was filled with 10% FBS medium. After 24 h, the cells were fixed with ethanol 100% and stained with crystal violet 0.5% in ethanol 20%. The cells in the chamber that did not invade were removed with a cotton swab. The invaded cells were photographed by an inverted microscope Olympus IX71 (Tokyo, Japan) and counted using ImageJ Fiji (NIH).

### 2.9 Gene Reporter

Gene reporter assay was performed to analyze the effects of STAT3 and APE1 redox inhibition on STAT3 transcriptional activity (Cardoso et al. 2012). To this aim, the cells were transfected with Lipofectamine 3000 reagent (ThermoFisher, L3000008). The plasmid p4xM67‐tk‐Luc was used to analyze STAT3 transcriptional activity, the 4xM67 pTATA TK‐Luc was a gift from Jim Darnell (Addgene plasmid # 8688; http://n2t.net/addgene:8688; RRID: Addgene_8688). pRLtk (Promega, E224A) was used as an internal control for luciferase. After 24 h of treatment, the cells transfected with p4xM67‐tk‐Luc were lysed with Dual‐Glo® Luciferase Assay System (Promega, E2920) to assess the luciferase activity. The luminescence was measuredusing a microplate reader EnVision XCite 2105 (Perkin Elmer).

### 2.10 Western blot Analysis

Total protein was extracted using 70 μl of RIPA lysis buffer containing Halt protease and phosphatase inhibitor cocktail 100× (ThermoFisher, 78440) and PhosSTOP (Roche, 4906845001). The protein quantification was performed using the Pierce BCA protein assay kit (ThermoFisher, 23225), prepared with 5x loading buffer (Lameli buffer), and boiled for 15 min at 90°C. 40 µg of protein was used to perform the electrophoresis on 8% SDS‐PAGE, transferred to PVDF membranes, and blocked with nonfat dry milk in Tris‐buffered saline with 0.1% of Tween (T‐TBS) for 1 h. The membranes were incubated with the following primary antibodies: anti‐APE1 (1:2000, MA1‐440, Invitrogen), anti‐STAT3 (1:1000, 12640, CellSignaling), anti‐Vinculin (1:10000, MA5‐11690, Invitrogen) and anti‐alpha‐tubulin (1:5000, T6199, Sigma‐Aldrich), followed by the respective secondary antibodies: anti‐Rabbit (1:5000, 31460, Invitrogen) and anti‐Mouse (1:5000, 31430, Invitrogen). The proteins were detected with SuperSignal™ West Pico PLUS Chemiluminescent Substrate (ThermoFisher, 34580). The band density analysis was carried out using ImageJ Fiji (McIlwain et al. 2018).

### 2.11 In silico data analysis

Data from breast cancer patients were obtained from TCGA (The Cancer Genome Atlas Program) to analyze gene expression based on mRNA levels and correlate these data with clinicopathological characteristics of breast cancer, such as the molecular subtypes. We analyzed female samples of primary tumors. The RNA‐seq data analysis was performed using the UCSC XENA platform (Goldman et al. 2020). Gene signatures were also used to evaluate the in‐silico association between APE1 redox (Shah et al. 2017) and STAT3 (Tan et al. 2019) activity, proliferation (Starmans et al. 2012), and metastasis (Mak et al. 2016) processes. The patients were grouped into high and low signature expression groups, according to 75% and 25% quartiles, respectively.

### 2.12 Statistical Analysis

The Kolmogorov‐Smirnov normality test evaluated the Gaussian distribution. The analysis with two groups was performed by the Mann‐Whitney test for non‐parametric data and the Student t‐test for parametric data. For multiple experimental groups, the analyses were performed using the Kruskal‐Wallis test followed by Dunn's posttest for non‐parametric data, and using ANOVA followed by Dunnett posttest for parametric data. The data were presented as mean ± standard deviation, and the results were considered statistically significant when *p* < 0.05. The data analysis was evaluated by GraphPad Prism 8.

## 3 Results

### 3.1 APE1 Redox Activity is Essential for STAT3 Transcriptional Activity

**Figure 2:**
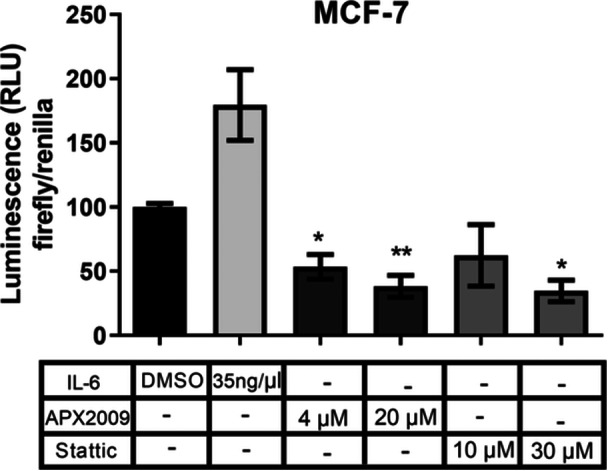
Transcriptional activity of STAT3 was assessed by Luciferase gene reporter assay with pM67 pTATA TK‐Luc, in MCF‐7 breast cancer cells. The cells were transfected with Lipofectamine 3000 reagent, treated with 4 and 20 μM of APX2009 and 10 and 30 μM of Stattic for 24 h and the transcriptional activity of STAT3 was evaluated by luciferase luminescence assay in a microplate reader. DMSO was used as vehicle control, and 35 ng/μL of IL‐6 for 30 min as a positive control of STAT3 induction. The normalization of luminescence was performed with pRLtk. The data are represented as means (%) ± Standard Deviation and considered statistically significant when *p* < 0.05 (*) or *p* < 0.01 (**) compared to DMSO. RLU = Relative Light Unit; DMSO = Dimethyl sulfoxide.

To investigate if the redox activity of APE1 can regulate STAT3 transcriptional activity, we performed a luciferase gene reporter assay using APX2009 and Stattic inhibitors. In MCF‐7, the treatment with 4 and 20 µM of the redox inhibitor (APX2009) decreased luminescence, suggesting a decrease in STAT3 transcriptional activity. In addition, 30 µM of Stattic also decreased STAT3 transcriptional activity (Figure 2). This suggests that the APE1 redox domain regulates STAT3 transcriptional activity.

Additionally, the protein levels of APE1 and STAT3 were evaluated using a Western blot assay. Our results showed that in both cell lines, MDA‐MB‐231 and MCF‐7, treatment with APX2009 and Stattic, individually or in combination, did not alter the protein levels of APE1 and STAT3 (supplementary Figures 1 and 2). These results demonstrate that APX2009 and Stattic inhibitors regulate the transcriptional activity of STAT3 without altering the protein levels of APE1 and STAT3.

### 3.2 APX2009 and Stattic Inhibitors Act Synergically for MDA‐MB‐231 and MCF‐7 Cells Cytotoxicity

**Figure 3:**
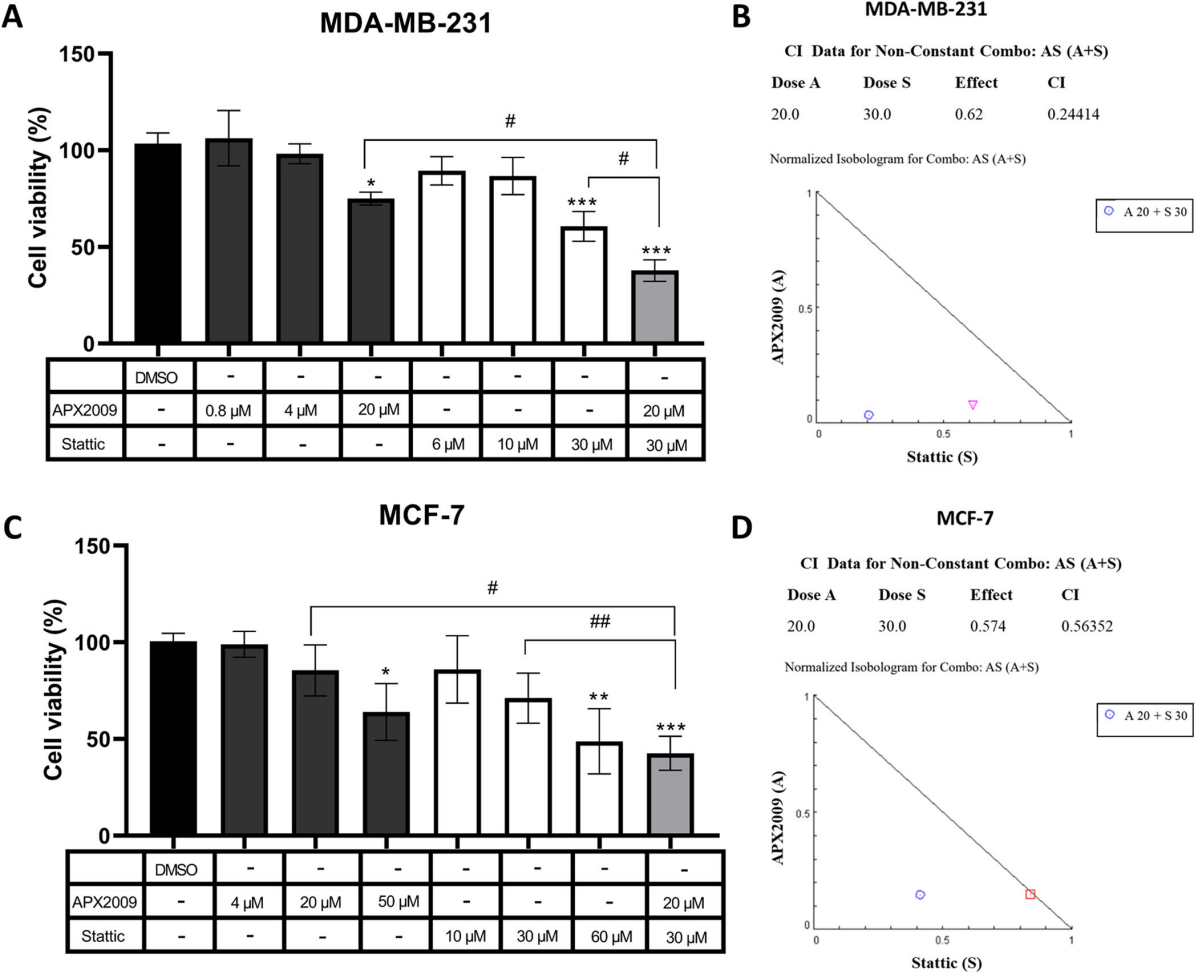
Viability of MDA‐MB‐231 (A) and MCF‐7 (C) breast cancer cells in response to APX2009 and Stattic. The cells were treated with 0.8, 4, 10, 20, and 50 μM of APX2009 and 6, 10, 30, and 60 μM of Stattic, individually or in combination, for 24 h, and the cell viability was measured by WST‐1 colorimetric assay in a microplate reader. DMSO was used as a vehicle control. The data are represented as means (%) ± Standard Deviation and considered statistically significant when *p* < 0.05 (*), *p* < 0.01 (**), and *p* < 0.001 (***) compared to DMSO. The comparison between the treatments was considered statistically significant when *p* < 0.05 (#) and *p* < 0.01 (##). The combination index was calculated for the combination of APX2009 (A) and Stattic (S) in MDA‐MB‐231 (B) and MCF‐7 (D) breast cancer cells. The effect represents the percentage of nonviable cells by WST‐1, and CI is the calculated value for the combination index by Compusyn software. DMSO = Dimethyl sulfoxide.

The WST‐1 assay was performed to investigate the effects of the treatment combination of APX2009 and Stattic in MCF‐7 and MDA‐MB‐231 breast cancer cells. The isolated treatment with 30 µM Stattic after 24 h decreased the viability of MDA‐MB‐231 cells, while a higher concentration of 60 µM decreased the viability of MCF‐7 cells. Similarly, MDA‐MB‐231 exhibited more sensitivity to APX2009 (10 µM) than MCF‐7 (50 µM). Additionally, cotreatment with 20 µM of APX2009 and 30 µM Stattic resulted in a greater decrease in cell viability in both cell lines compared to the isolated treatments, suggesting that the combination of inhibitors is more effective in promoting cytotoxicity in breast cancer cells (Figure 3).

To analyze the interaction between APX2009 and Stattic, we calculated the combination index of the inhibitors in breast cancer cell lines. The results indicated synergism between the two inhibitors in both cell lines (Figure 3), and suggested that Stattic and APX2009 act selectively in tumor cells, as the selectivity index (SI) is higher than 1 for both inhibitors, comparing the IC50 values of tumor and nontumor cells (Supplementary Figure 3).

### 3.3 APX2009 and Stattic Act Synergically for MDA‐MB‐231 and MCF‐7 Cells Proliferation Inhibition

**Figure 4:**
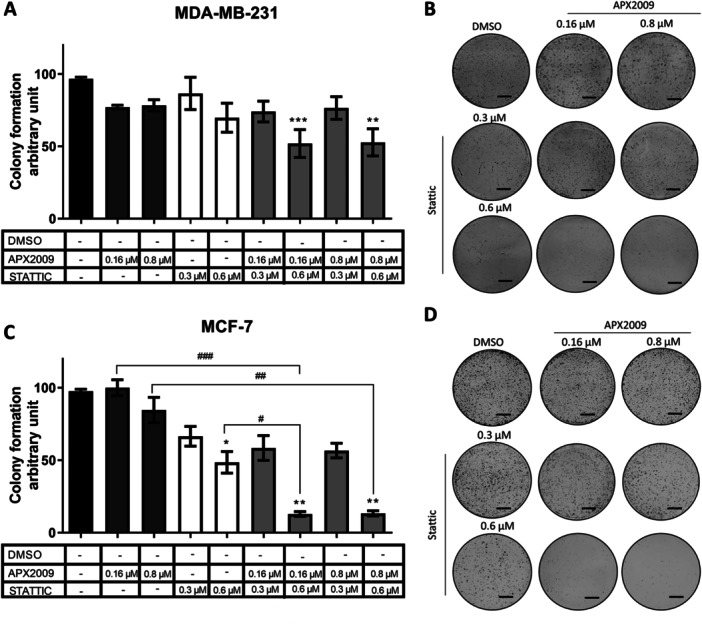
Representative images of clonogenic assay of MDA‐MB‐231 (A and B) and MCF‐7 (C and D) breast cancer cells in response to treatment with 0.16 and 0.8 μM of APX2009 and 0.3 and 0.6 μM of Stattic, individually or in combination. The cells were seeded in 6‐well plates at low density (4 × 10³ cells/well for MDA‐MB‐231 and 8 × 10³ cells/well for MCF‐7). After 4 h, the cells were treated with 0.3 and 0.6 µM of Stattic, 0.16 and 0.8 µM of APX2009, and the combination of 0.3 and 0.6 µM of Stattic + 0.16 and 0.8 µM of APX2009. After 7 days, the colonies were stained with crystal violet, and the absorbance was measured with a microplate reader. DMSO was used as a vehicle control. The data are represented as means (%) ± Standard Deviation and considered statistically significant when *p* < 0.05 (*), *p* < 0.01 (**), and *p* < 0.001 (***) compared to DMSO. Scale bar = 5000 μm. DMSO = dimethyl sulfoxide.

We performed the anchorage‐dependent colony formation assay to investigate the effects of APX2009 and Stattic combination on cell proliferation. The Stattic concentrations of 0.3 and 0.6 µM, and 0.16 and 0.8 µM of APX2009, did not alter the colony formation in MDA‐MB‐231. However, the combination of 0.16 or 0.8 µM of APX2009 with 0.6 µM of Stattic led to decreased colony formation (Figure 4). For MCF‐7, the treatment with both inhibitors at 0.16 and 0.6 µM also led to a higher decrease in colony formation than isolated treatments (Figure 4). This data reinforced the synergism hypothesis between APE1 and STAT3 also on the proliferation of cancer cells.

### 3.4 The Treatment With APX2009 and Stattic Induces Cell Death by Apoptosis

**Figure 5:**
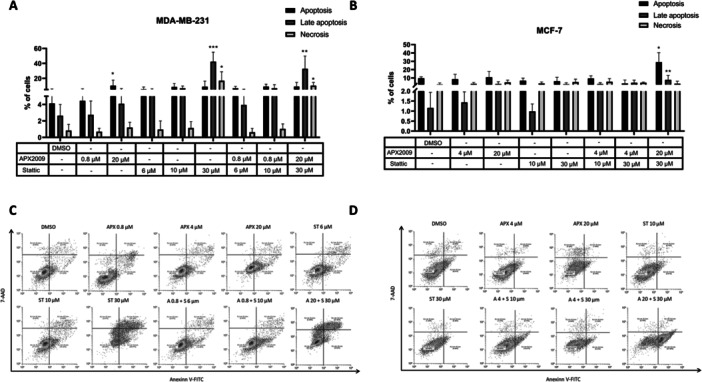
Analysis of cell death performed by flow cytometry in MDA‐MB‐231 (A) and MCF‐7 (B) breast cancer cells in response to treatment with 0.8, 4, and 20 μM of APX2009 and 6, 10, and 30 μM of Stattic, individually or combined, for 24 h. DMSO was used as a vehicle control. Representative analysis of cells stained with Annexin V‐FITC and 7‐AAD (C and D). Apoptosis was analyzed by the number of cells positive for Annexin and negative for 7‐AAD, late apoptosis by cells positive for both markers, and necrosis by cells positive for only 7‐AAD. The data are represented as means (%) ± Standard Deviation and considered statistically significant when *p* < 0.05 (*), *p* < 0.01 (**), and *p* < 0.001 (***) compared to DMSO. DMSO = dimethyl sulfoxide.

To identify the mechanism by which the treatments led to decreased cell viability, we performed AnnexinV/7‐AAD staining by flow cytometry. For MDA‐MB‐231, the treatments with 20 µM of APX2009 increased the percentage of cells stained with Annexin compared to the control, suggesting apoptosis. Moreover, treatment with 30 µM Stattic increased the percentage of cells stained with Annexin and 7‐AAD, suggesting cell death by late apoptosis and necrosis. The combination of 30 µM of Stattic and 20 µM of APX2009 also induced late apoptosis and necrosis in MDA‐MB‐231 (Figure 5). For MCF‐7, treatment with APX2009 or Stattic did not induce cell death. However, the combination of 30 µM Stattic and 20 µM APX2009 increased the percentage of cells stained with Annexin and 7‐AAD, suggesting cell death by apoptosis and late apoptosis (Figure 5).

### 3.5 The Combination of APX2009 and Stattic is More Potent Than the Isolated Treatment in Reducing Cell Migration and Invasion of MDA‐MB‐231 and MCF‐7 Cell Lines

**Figure 6:**
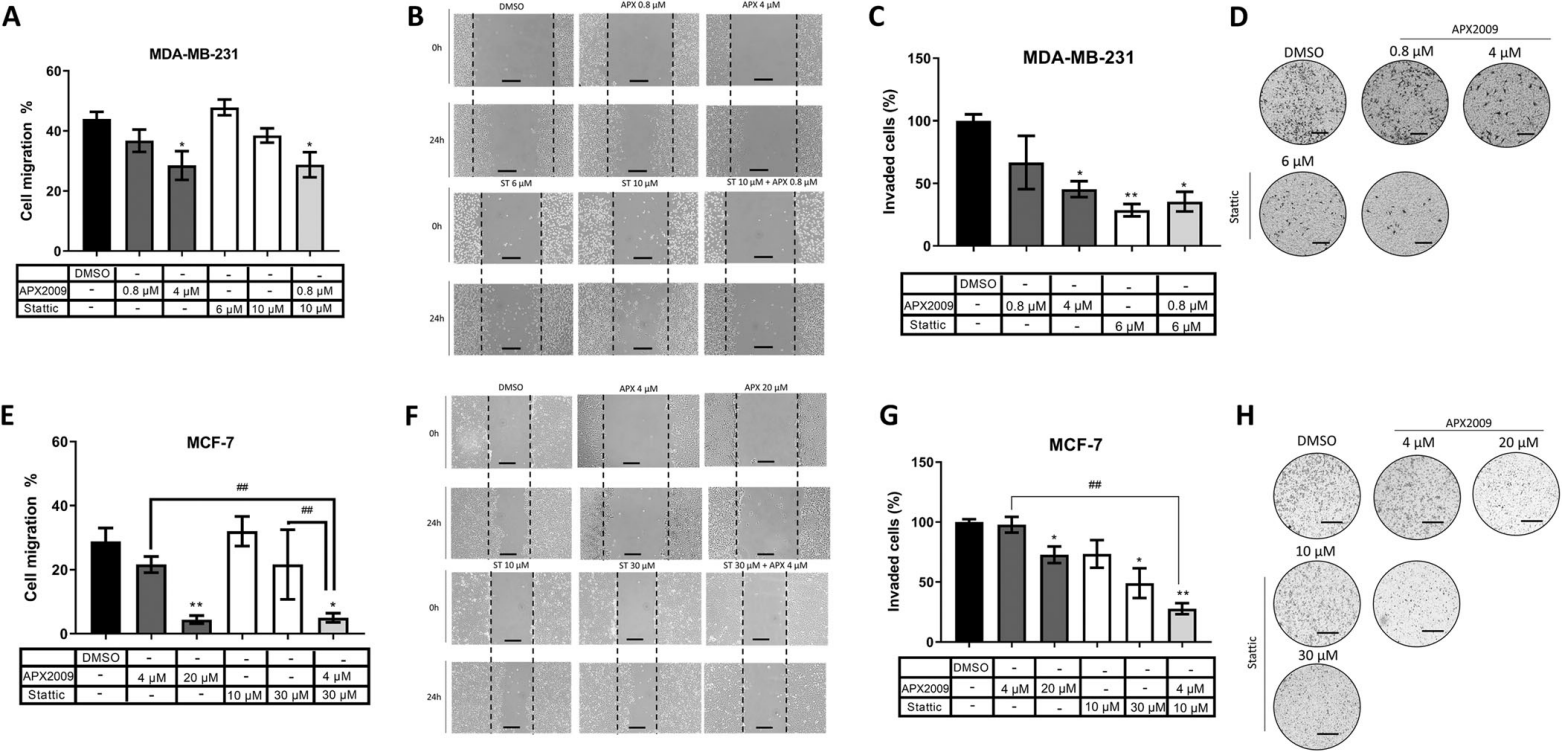
Analysis of cell migration by Wound Healing assay and cell invasion by Matrigel Transwell assay, in MDA‐MB‐231 (A, B, C and D) and MCF‐7 (E, F, G and H) breast cancer cells after treatment with 0.8, 4, and 20 μM of APX2009 and 6, 10, and 30 μM of Stattic, individually or combined, for 24 h. DMSO was used as a vehicle control. The data are represented as means (%) ± Standard Deviation, and considered statistically significant when *p* < 0.05 (*), *p* < 0.01 (**) compared to DMSO. DMSO = Dimethyl sulfoxide. The comparison between the treatments was considered statistically significant when *p* < 0.01 (##). Scale bar = 200 μm.

To investigate the roles of APE1 and STAT3 in the aggressive phenotype, we performed Wound Healing and Matrigel Transwell Invasion assays using APX2009 and Stattic inhibitors. In the MDA‐MB‐231 cell line, the treatment with Stattic and APX2009 separately did not alter cell migration. However, the combination of 0.8 µM of APX2009 and 10 µM of Stattic was potent in decreasing cell migration (Figure 6). Similarly, the combination of 30 µM Stattic and 4 µM APX2009 decreased MCF‐7 cell migration (Figure 6). Both APX2009 and Stattic decreased the cell invasion of MDA‐MB‐231 and MCF‐7. In addition, combining these two treatments further reduced the invasiveness of MCF‐7 cells, using 4 µM of APX2009 and 10 µM of Stattic (Figure 6).

### 3.6 Redox APE1 and STAT3 Gene Signatures Are Positively Correlated in Breast Cancer Samples of TCGA

**Figure 7:**
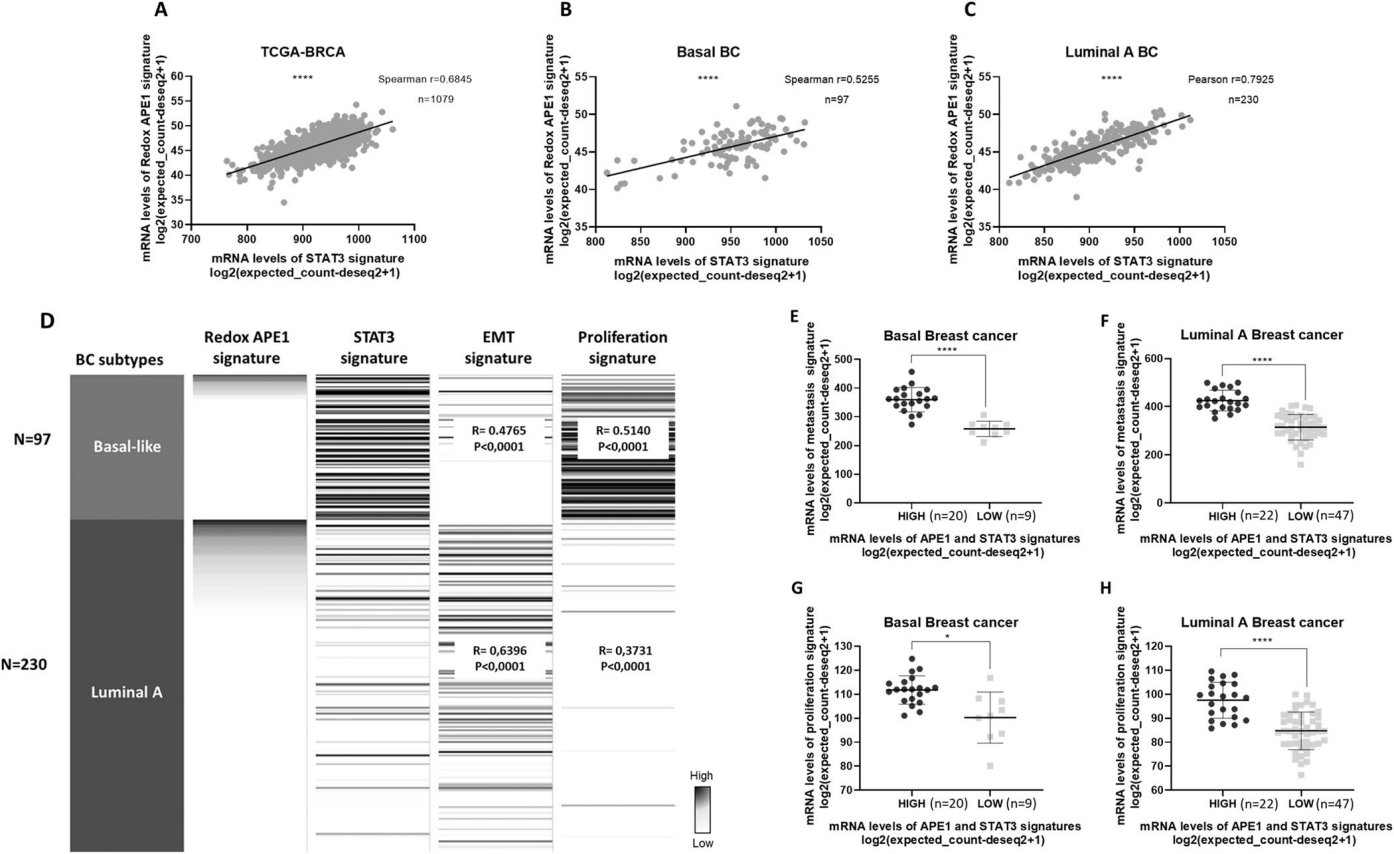
Correlation of APE1 redox activity signature with STAT3 signature in TCGA breast cancer patient samples from XENA platform (A), and in the basal (B) and luminal A (C) breast cancer subtypes. The data was considered statistically significant when *p* < 0.0001 (****). Correlation of APE1 redox activity signature and STAT3 signature with metastasis and proliferation signatures in TCGA breast cancer (BC) patient samples of basal and luminal A subtypes (D). mRNA levels of metastasis and proliferation signatures in breast cancer patient samples of TCGA with high and low levels of both APE1 redox activity and STAT3 signatures. The breast cancer samples were divided into basal (E and G) and luminal A (F and H) subtypes. The correlations were considered weak when 0.39<r > 0.2, moderate when 0.69<r > 0.4, and strong when 0.89<r > 0.7.

To clinically investigate the association between the redox activity of APE1 and STAT3 found in vitro, we analyzed the correlation of the expression of the APE1 redox activity gene signature with the STAT3 signature in the breast cancer patients' samples in the TCGA Target Gtex database on the UCSC Xena platform. The results demonstrated a moderate and strong positive correlation between these signatures, in basal (*r* = 0.5255) and luminal A (*r *= 0.7925) subtypes, respectively, suggesting a positive association between redox activity of APE1 and STAT3 in breast cancer (Figure 7).

### 3.7 Redox APE1 and STAT3 Gene Signatures Are Positively Correlated With Proliferation and Metastasis Signatures in Breast Cancer Samples of TCGA

The in vitro results showed the participation of redox APE1 and STAT3 in cellular processes essential to cancer, such as proliferation and aggressiveness. Thus, we investigated the correlation between the expression of APE1 redox domain and STAT3 signatures, with proliferation and metastasis signatures. The analyses were performed using samples of luminal A and basal breast cancer subtypes, represented by MCF‐7 and MDA‐MB‐231 cell lines, respectively. We found that APE1 redox and STAT3 signatures are positively correlated with proliferation and metastasis signatures in basal (metastasis *r* = 0.4765 *p* < 0.0001; proliferation *r* = 0.5140 *p* < 0.0001) and luminal A (metastasis *r* = 0.6396 *p* < 0.0001; proliferation *r* = 0.3731 *p* < 0.0001) subtypes (Figure 7). In addition, patients with high expression of redox APE1 and STAT3 signatures concomitantly also exhibited high levels of proliferation and metastasis signatures in basal and Luminal A subtypes (Figure 7).

## 4 Discussion

Despite advances in cancer treatment, many tumors still become resistant to conventional therapies (Lin‐Rahardja et al. 2023). Therefore, it is essential to identify new proteins that could be used as therapeutic targets against cancer. Some studies have demonstrated that combining different compounds could be more effective in cancer treatment, decreasing cell proliferation and aggressiveness (Lin‐Rahardja et al. 2023).

In this context, both APE1 and STAT3 proteins have been listed as possible targets (Banerjee and Resat 2016; Wen et al. 2016), and the combined inhibition of these proteins led to a decrease in cancer cell survival in pancreatic and bladder cancer (Caston et al. 2021). However, the effect of dual inhibition of APE1 redox activity and STAT3 in breast cancer proliferation, survival, and aggressiveness is still unknown.

Previous studies showed that the APE1 redox domain can activate STAT3 transcriptional function in pancreatic cancer (Cardoso et al. 2012). Nevertheless, this interaction had not yet been described in breast cancer. In our study, we first reported that APX2009 reduces STAT3 transcriptional activity without affecting its protein levels, suggesting that the redox domain of APE1 also regulates STAT3 in breast cancer. Moreover, we demonstrated that the combined inhibition of redox APE1 and STAT3, using APX2009 and Stattic, respectively, was more effective in decreasing the viability of MDA‐MB‐231 and MCF‐7 breast cancer cells than the individual treatment, highlighting a synergistic effect between these two compounds. Although APX2009 and Stattic alone induce cell death in bladder and pancreatic cancer, as observed by Fishel et al. 2019 and Guo et al. 2022a, respectively, our findings reveal that their combination is even more potent than individual treatment in inducing cell death in MCF‐7 cells. In addition, we showed that the combination of these inhibitors synergistically decreases the clonogenic potential of breast cancer cells, an effect not previously reported. Together, these results corroborate our hypothesis of a positive association between redox APE1 and STAT3 in breast cancer.

The ability of cancer cells to migrate and invade is the main characteristic of metastasis, which is directly related to tumor aggressiveness (Valastyan and Weinberg 2011). Previously, our group demonstrated that APX2009 decreased breast cancer cell migration and invasion (Siqueira, Rodrigues, et al. 2024). In this scenario, we reported that the combined inhibition of redox APE1 and STAT3 was more effective in decreasing the migration of MCF‐7 and MDA‐MB‐231 cancer cells compared to individual treatment. Cardoso et al. 2012 also observed a decrease in the migration of pancreatic cancer cells after treatment with APE1 and STAT3 inhibitors. Furthermore, there are no reports in the literature on the combined effect of inhibitors of the redox domain of APE1 and STAT3 on the invasive potential of tumor cells. Therefore, we showed for the first time that dual targeting of redox APE1 and STAT3 could decrease the cell invasion of MCF‐7 breast cancer cells. Altogether, these results suggested that combined treatments, such as the combination of APX2009 and Stattic, could be an important approach against cancer aggressiveness.

To compare the data obtained in vitro with data from patients with the corresponding breast cancer subtypes in vitro (luminal A, and basal), we used public TCGA data available on the Xena platform. Therefore, we can evaluate the clinical impact of the target proteins, APE1 and STAT3. In silico data showed that breast cancer patients of TCGA exhibited a positive correlation between the gene expression of the APE1 redox function signature and the STAT3 signature, which corroboratesour in vitro findings. These signatures are also positively correlated with proliferation and metastasis signatures, suggesting again that the redox function of APE1 and STAT3 co‐participate in regulating cellular processes in cancer. It has already been demonstrated that APE1 and STAT3 alone can regulate these processes. Yang et al. 2018 observed that overexpression of APE1 induced metastasis‐associated processes, such as epithelial‐mesenchymal transition, and the redox inhibition of APE1 reversed this process. Moreover, STAT3 can activate different genes that participate in the metastasis process, such as metalloproteinases, Vimentin, and Fascin (Carpenter and Lo 2014). However, the combined signaling of these proteins has not been described before, which suggests the co‐participation of APE1 and STAT3 in regulating the aggressiveness in breast cancer.

There are already clinical studies aiming to inhibit APE1 in pancreatic, prostate, bladder, ovary, and other types of cancer (NCT0337508), and STAT3 (NCT00955812) in leukemia (Chu et al. 2019; Hayakawa et al. 2013). We further emphasize that APX2009, used in this study, has been demonstrated to be more effective in reducing cell viability, migration, and invasion of breast cancer cells when compared to the inhibitor APX3330 (Guerreiro et al. 2017). Therefore, our findings, along with new studies focusing on APE1 and STAT3, could support identifying new promising pharmacological inhibitors for these proteins and contribute to the development of enhanced therapeutic strategies against cancer, including breast cancer.

## 5 Conclusion

Our findings demonstrated that the APE1 redox inhibitor APX2009 impaired STAT3 transcription activity. To the best of our knowledge, this is the first demonstration that dual targeting of redox APE1 and STAT3 in breast cancer is a more effective strategy than single inhibitors for reducing cell survival and proliferation, inducing cell death, and reducing cell aggressiveness through the decrease in cell migration and invasion. Therefore, the APE1 redox domain and STAT3 appear promising targets for a new possible combined treatment, which could be a more efficient strategy against breast cancer.

## Author Contributions

**Mariana Moreno de Sousa Rodrigues:** writing – original draft, methodology, investigation, formal analysis, visualization. **Priscyanne Barreto Siqueira:** methodology, investigation**. Ana Clara Cavallo Dobao:** investigation. **Maria Eduarda Barbosa Mourão:** Investigation. **Bruno Ricardo Barreto Pires:** writing – review and editing. **Adenilson de Souza da Fonseca:** funding acquisition. **Ísis Salviano Soares de Amorim:** supervision, project administration, writing – review and editing. **Andre Luiz Mencalha:** conceptualization, supervision, project administration, writing – review and editing, funding acquisition.

## Conflicts of Interest

The authors declare no conflicts of interest.

## Supporting information


**Figure S1:** Protein levels of APE1 and STAT3 in MDA‐MB‐231 breast cancer cells after treatment with APX2009 and Stattic, individually or combined. (A) Representative images. (B) The data from three experiments are represented as means (%) ± SD. DMSO was used as a vehicle control. Vinculin was used as a loading control for Western blot normalization. APX = APX2009; ST = Stattic. **Figure S2:** Protein levels of APE1 and STAT3 in MCF‐7 breast cancer cells after treatment with APX2009 and Stattic, individually or combined. (A) Representative images. (B) The data from three experiments are represented as means (%) ± SD. DMSO was used as a vehicle control. Tubulin was used as a loading control for Western blot normalization. APX = APX2009; ST = Stattic. **Figure S3:** MCF10A cells viability after 24 hours of treatment with 4, 10, 20, and 50 μM of APX2009 and 6, 10, 30, and 60 μM Stattic, individually or combined. DMSO was used as a vehicle control. The data are represented as means (%) ± SD and considered statistically significant when *p* < 0.05 (*), *p* < 0.01 (**) compared to DMSO.

## Data Availability

Data sharing not applicable to this article as no datasets were generated or analysed during the current study.
