# Supplementary material for: Synergistic Reduction of Breast Cancer Cell Viability and Aggressiveness Through Dual Inhibition of APE1 Redox Function and STAT3 Signaling
Source: Cell Biol Int. 2025 Oct 15;49(12):1787–98. doi: 10.1002/cbin.70094 (PMC12605823; doi:10.1002/cbin.70094)
Supplement: Supplementary file 1 — Figure S1: Protein levels of APE1 and STAT3 in MDA‐MB‐231 breast cancer cells after treatment with APX2009 and Stattic, individually or combined. (A) Representative images. (B) The data from three experiments are represented as means (%) ± SD. DMSO was used as a vehicle control. Vinculin was used as a loading control for Western blot normalization. APX = APX2009; ST = Stattic. Figure S2: Protein levels of APE1 and STAT3 in MCF‐7 breast cancer cells after treatment with APX2009 and Stattic, individually or combined. (A) Representative images. (B) The data from three experiments are represented as means (%) ± SD. DMSO was used as a vehicle control. Tubulin was used as a loading control for Western blot normalization. APX = APX2009; ST = Stattic. Figure S3: MCF10A cells viability after 24 hours of treatment with 4, 10, 20, and 50 μM of APX2009 and 6, 10, 30, and 60 μM Stattic, individually or combined. DMSO was used as a vehicle control. The data are represented as means (%) ± SD and considered statistically significant when p < 0.05 (*), p < 0.01 (**) compared to DMSO. [file CBIN-49-1787-s001.docx]

**Supplementary figures**

**APE1 and STAT3 protein levels in MDA-MB-231 and MCF-7 cells using a western blot assay.**

Our results showed that in both cell lines, MDA-MB-231 (Fig. S1) and MCF-7 (Fig. S2), treatment with APX2009 and Stattic, individually or in combination, did not alter the protein levels of APE1 and STAT3.


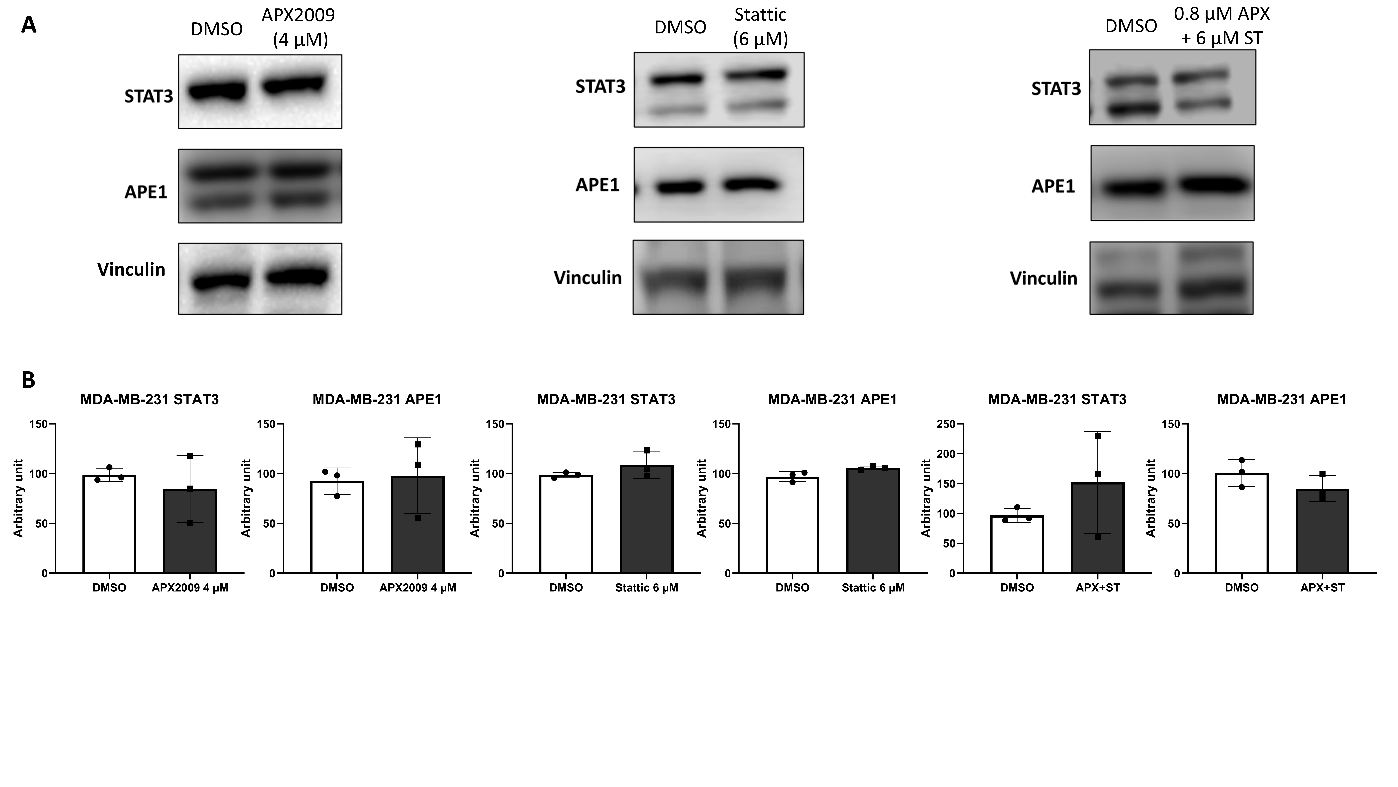


Figure S1. Protein levels of APE1 and STAT3 in MDA-MB-231 breast cancer cells after treatment with APX2009 and Stattic, individually or combined. (A) Representative images. (B) The data from three experiments are represented as means (%) ± SD. DMSO was used as a vehicle control. Vinculin was used as a loading control for Western blot normalization. APX = APX2009; ST = Stattic.


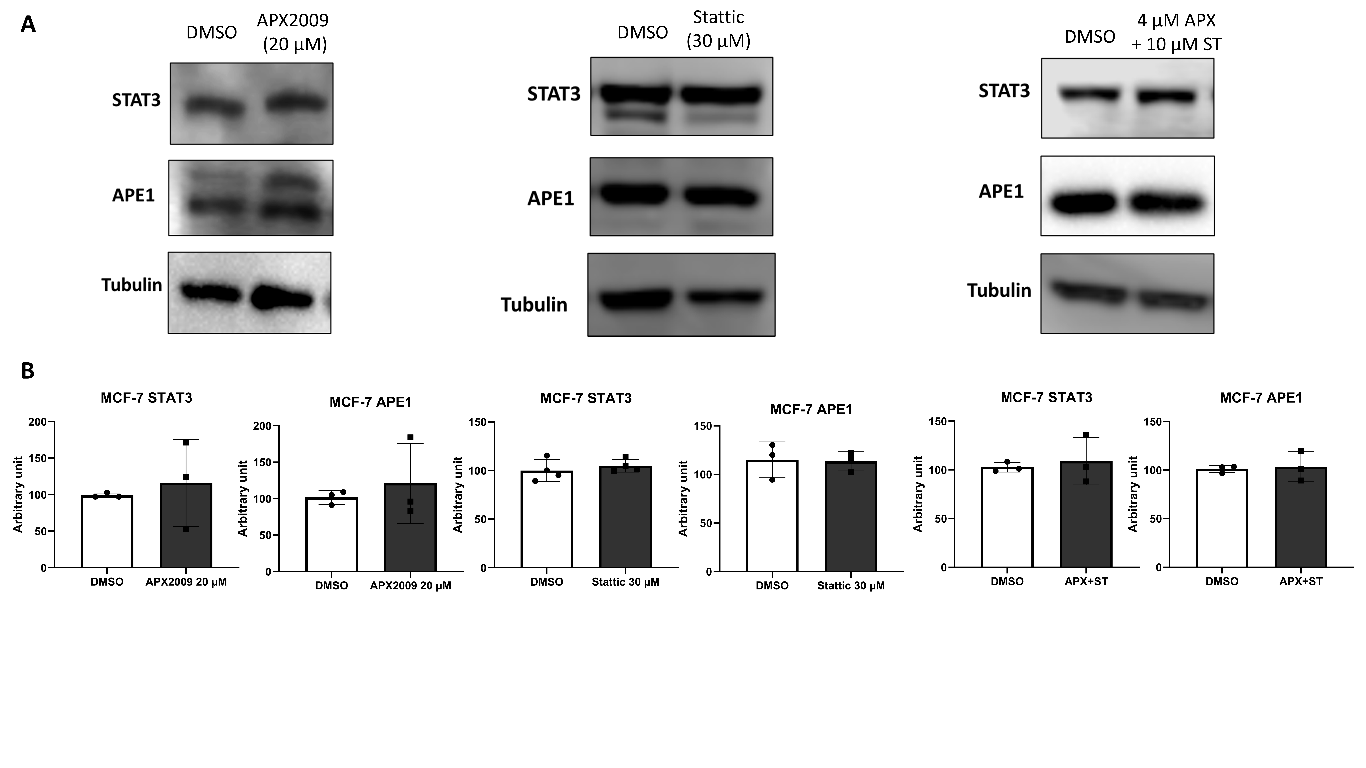


Figure S2. Protein levels of APE1 and STAT3 in MCF-7 breast cancer cells after treatment with APX2009 and Stattic, individually or combined. (A) Representative images. (B) The data from three experiments are represented as means (%) ± SD. DMSO was used as a vehicle control. Tubulin was used as a loading control for Western blot normalization. APX = APX2009; ST = Stattic.

**Stattic and APX2009 in MCF10A non-tumor cells viability**

Our results suggest that Stattic and APX2009 act selectively in tumor cells, as the selectivity index (SI) is higher than 1 for both inhibitors, comparing the IC50 values of tumor and non-tumor cells. Formula used: SI = (IC50 of MCF10A cells) / (IC50 of MDA-MB-231 or MCF-7 cells) (Silva et al., 2021).

Figure S3. MCF10A cells viability after 24 hours of treatment with 4, 10, 20, and 50 μM of APX2009 and 6, 10, 30, and 60 μM Stattic, individually or combined. DMSO was used as a vehicle control. The data are represented as means (%) ± SD and considered statistically significant when p<0.05 (*), p<0.01 (**) compared to DMSO.
